# Constructing Lithium-Free Anode/Separator Interface via 3D Carbon Fabric Scaffold for Ultrasafe Lithium Metal Batteries

**DOI:** 10.34133/research.0267

**Published:** 2023-11-08

**Authors:** Dongdong Li, Shengchen Yang, Zijian Zheng, Wen-Yong Lai

**Affiliations:** ^1^ State Key Laboratory of Organic Electronics and Information Displays (SKLOEID), Institute of Advanced Materials (IAM), School of Chemistry and Life Sciences, Nanjing University of Posts and Telecommunications, 9 Wenyuan Road, Nanjing 210023, China.; ^2^ Department of Materials Chemistry, Huzhou University, 1 Xueshi Road, Huzhou 313000, China.; ^3^ Laboratory for Advanced Interfacial Materials and Devices, School of Fashion and Textiles, Department of Applied Biology and Chemical Technology, Research Institute for Intelligent Wearable Systems (RI-IWEAR), Research Institute for Smart Energy (RISE), The Hong Kong Polytechnic University, Hong Kong SAR 999077, China.

## Abstract

Metallic lithium represents a promising anode candidate to be utilized in future high-energy lithium batteries. However, the undesirable dendrite growth and fragile solid-electrolyte interphase (SEI) pose critical challenge for pursuing further practical application. In contrast to traditional approaches of using inert/lithiophilicity coating, here, we demonstrate a reverse strategy of introducing a highly conductive and lithophobic carbon fabric (CF) scaffold on lithium foil to guide a favorable nucleation site of lithium far away from the anode/separator interface. The CF scaffold with high conductivity can couple with inner electric field for achieving a uniform distribution of the lithium-ion flux, while the lithophobic feature offers the condition to guide the preferred deposition of lithium onto the underlying lithium foil, which greatly reduces the risk of dendrite-induced short circuits. Moreover, the SEI immersed in the CF scaffold is well supported by CF fibers and therefore exhibits extremely high stability during charge–discharge cycles. As a result, the lithium/CF anodes show >2,000-h stable cycling at 0.5 mA cm^−2^. Lithium metal batteries equipped with our lithium/CF anode deliver a high capacity retention of ~99.99% per cycle, i.e., retain ~97.3% capacity after 200 cycles. The unique interface-regulation strategy is versatile for various conductive scaffolds (e.g., ultrathin and ultralight conductive fabrics), exhibiting high superiority for highly safe lithium metal batteries.

## Introduction

**Fig. 1.**
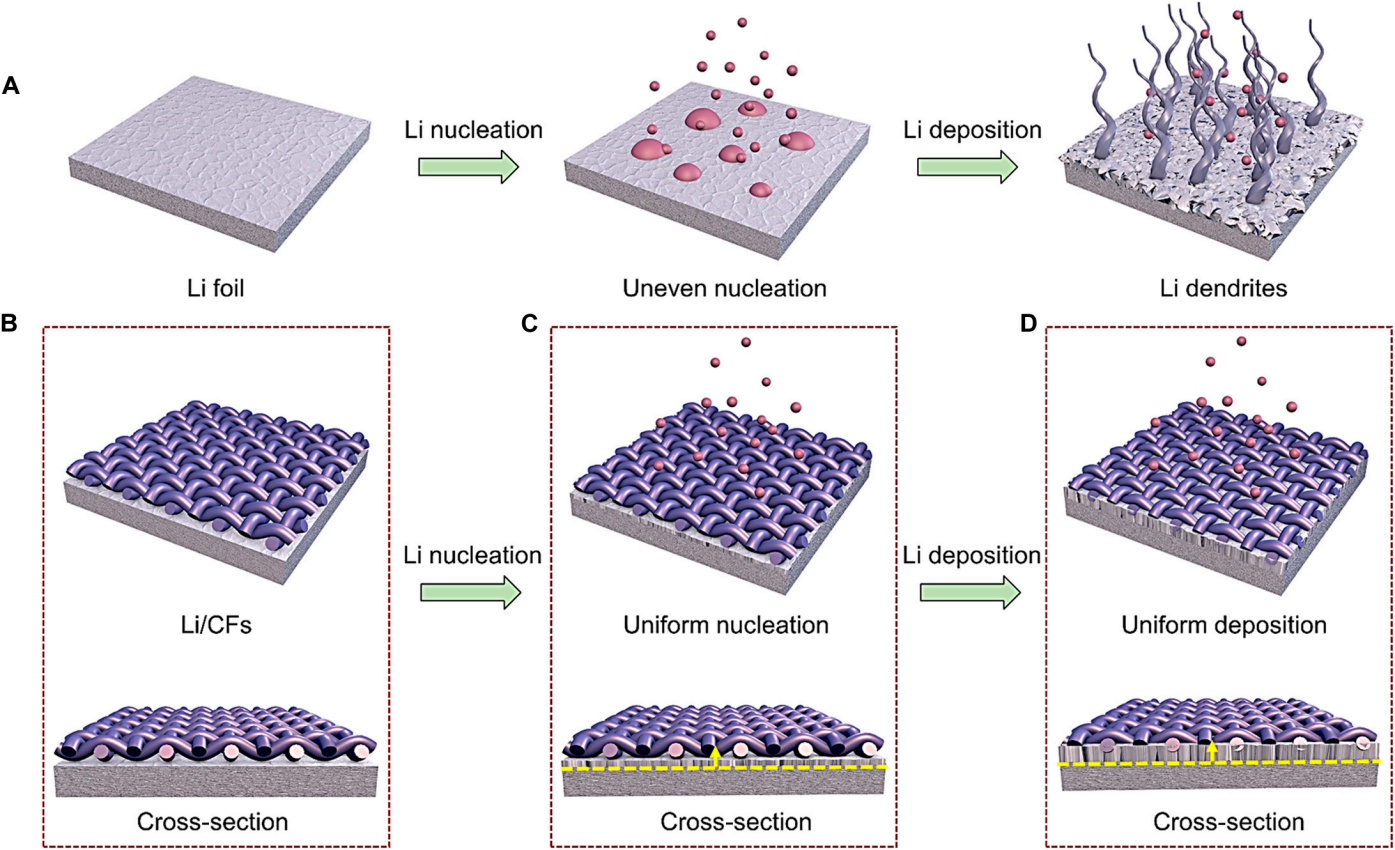
Schematic illustration of Li deposition on Li-foil and Li/CFs anodes. (A) Uneven nucleation and dendric Li growth on bare Li foil. (B to D) Uniform Li deposition toward the bottom of the lithophobic 3D CF scaffold. The yellow arrows in (C) and (D) represent the growth direction of Li.

Emerging electronic markets such as intelligent displays, roll-up phones, bioelectronics, and electric vehicles require the development of efficient energy power as an alternative to current low-capacity lithium (Li)-ion batteries for long-term endurance [1–8]. Metallic Li, which can provide ultrahigh theoretical capacity and extremely low electrode potential, represents a promising anode choice for ascendant Li metal battery (LMB) systems [9–13]. However, the critical safety challenge, i.e., dendritic Li growth during charge–discharge cycles, may trigger the internal short circuits via separator piercing, which largely increases the risks of battery fire and explosion (Fig. 1A) [14–23]. Another major problem is that the fragile solid-electrolyte interphase (SEI) that spontaneously builds on anode surface could be easily destroyed during the volume change of Li, which can further induce a low Coulombic efficiency (CE) and poor cyclic stability [24–27]. As such, it is desirable to develop approaches of achieving dendrite-free Li metal anode and reinforced SEI for safer and reliable LMBs.

Among various efforts of suppressing Li dendrites, several important strategies including modifying organic electrolytes [28], designing artificial SEI layers [29,30], developing solid-state electrolytes [31], and especially constructing 3-dimensional (3D) current collectors represent efficient progress for achieving high-performance LMBs [32–34]. With using 3D current collectors, dendric Li growth could be markedly suppressed by using the reduced local current density [35]. Meanwhile, the porous framework structure with plenty of internal space could alleviate the large volume change of Li during battery cycling [32]. However, short circuits still occur along the interface between anode and separator during long-term charge–discharge cycles. The deadliest factor that causes this trouble is the preferential top deposition of Li on 3D current collectors [36,37]. Therefore, regulating the original nucleation location of Li, i.e., guiding a favorable nucleation site of Li far away from the anode/separator interface, is critical yet remains to be studied in depth for achieving long-life and safe LMBs.

In our previous works, an inverted Li metal anode via flipping a lithiated carbon fabric (CF) as well as an Au-coated CF as Janus current collector were demonstrated to improve the electrochemical properties of Li anodes by architectural design of electrodes [38,39]. The bottom Li metal or Au layer could be used to induce a favorable bottom nucleation of Li, which greatly improved the safety and stability of LMBs. However, the fabrication of anodes employed lab-level electrodeposition method with limited scalable capability, claiming for new and simple techniques that compatible with industrial production.

Li deposition is a diffusion-controlled kinetic process. This feature promotes the traditional strategy of using an electronic inert/lithiophilic coating to realize a homogeneous deposition of Li, such as the construction of a self-assembled molecule monolayer [40], a sponge-driven elastic interface [41], electrical conductivity gradient heterofibrous scaffolds [42], and a nanoscale inorganic–organic coating [43]. These inert coatings can passivate Li metal anodes with a reduced probability of side reactions [44], while these lithiophilic modifications provide continuous channels for Li-ion migration, which lead to a reduced nucleation barrier for homogeneous Li nucleation/growth [45]. However, these electronic inert coatings cannot be coupled with the vertical electrical field inside Li batteries due to their low conductivity and thus inefficient to achieve uniform distribution of Li-ion flux in these 3D structures. As an alternative option, constructing conductive scaffolds represents a promising approach to realize a homogeneous Li nucleation/deposition by regulating the vertical electrical field. However, electrically active scaffolds may further promote the deposition of Li along the anode/separator interface, attributing to the reduced nucleation barrier with increasing conductivity [42], which boosts the risk of dendrite-induced short circuits. Therefore, regulating the deposition route of Li by using electrically active scaffolds still need efforts greatly.

Here, we first propose an unprecedentedly efficient electric field-regulating strategy to construct a favorable Li-free anode/separator interface by using a highly conductive and lithophobic CF scaffold (Fig. 1B), which represents a new technique of suppressing dendric Li growth on anode surface. Different from traditional inert/lithiophilicity coatings, the CF scaffold with high conductivity can be coupled with inner electric field to achieve a highly homogeneous Li-ion flux, whereas the lithophobic feature promotes the favorable deposition of Li onto the underlying Li foil (Fig. 1C). The resulting Li/CF anodes demonstrate excellent cyclic stability with low-voltage polarization at the current densities of 0.5, 1, and 5 mA cm^−2^ and the capacities of 1, 3.5, and 5 mAh cm^−2^. The robust CFs above Li foil can be also used as a porous skeleton to alleviate the volume change of Li and reinforce the fragile SEI layer during repeated charge–discharge cycles (Fig. 1D). LMBs equipped with the Li/CF anodes exhibit remarkable charge–discharge properties with ~97.3% capacity retention after 200 cycles. Moreover, the highly conductive and lithophobic scaffolds can be extended to other conductive films (e.g., ultrathin conductive fabrics), demonstrating effectiveness of the unique anode configuration for safer LMBs.

## Results

**Fig. 2.**
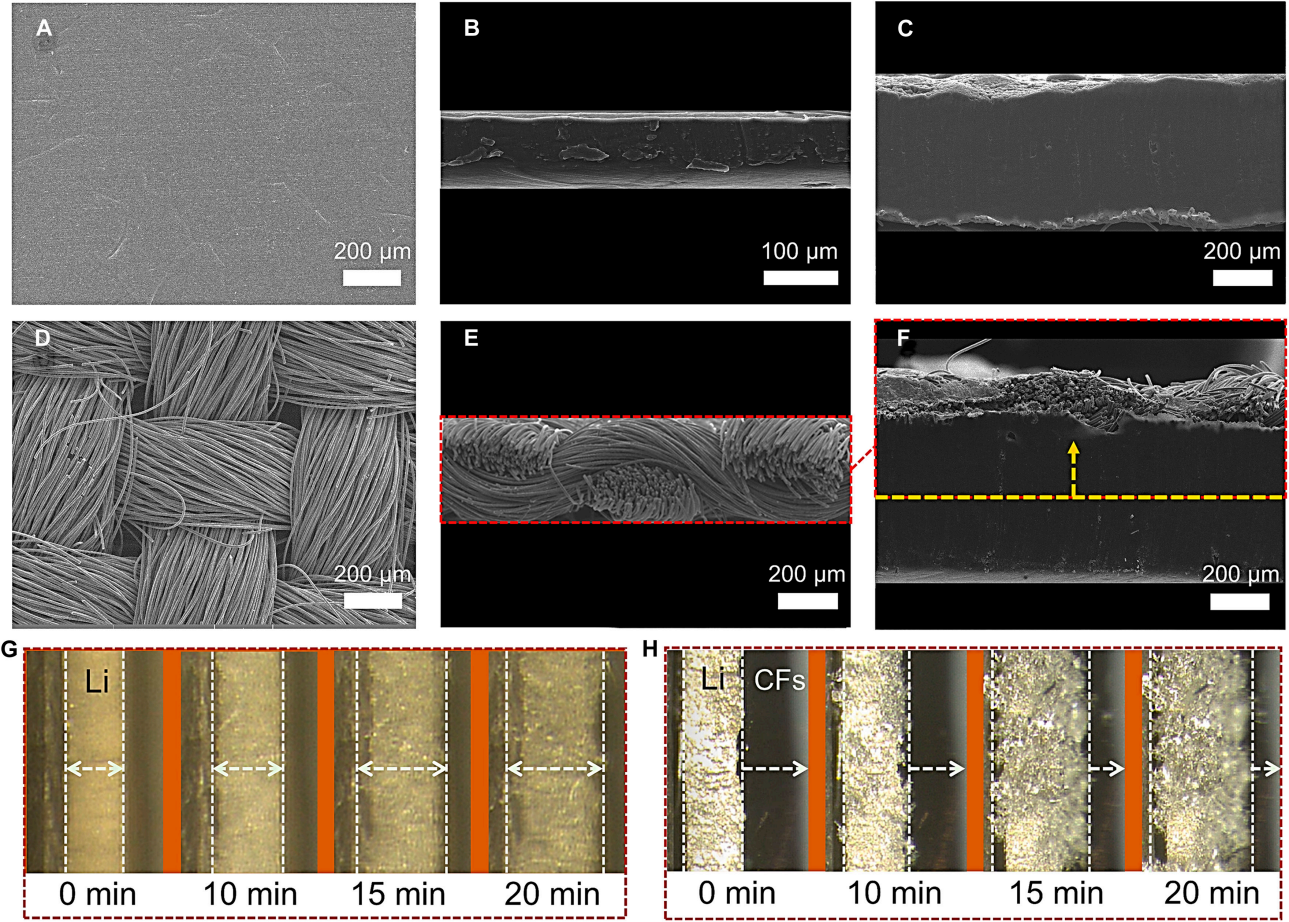
Li plating behaviors on Li-foil and Li/CF anodes. (A and B) SEM images of the top and cross-section of Li foil. (C) Cross-sectional SEM image of Li foil after Li plating (30 mAh cm^−2^). (D and E) SEM images of the top and cross-section of CFs. (F) Cross-sectional SEM image of Li/CF anode after Li plating (30 mAh cm^−2^). Optical microscopy images of the (G) Li-foil and (H) Li/CF anodes with increasing Li plating time. The arrows in (F) to (H) represent the growth direction of Li.

As shown in Fig. 1B, the Li/CF anode can be easily prepared by overlying a layer of CF scaffold on a bare Li foil (areal capacity: 15 mAh cm^−2^). The detailed preparation procedure can be found in Materials and Methods. Figure 2A and B exhibits the scanning electron microscopy (SEM) images of a bare Li foil. The surface morphology is very smooth except for some tiny wrinkles by carefully inspecting the microimages. After a high-capacity Li plating (30 mAh cm^−2^), we can observe some noticeable convex and concave structures with a large volume expansion (Fig. 2C), which is regarded as the main factor of inducing dendric Li growth and the fragile SEI layer. Figure 2D and E presents the surface and cross-sectional SEM images of the lithophobic CF scaffold on the surface of Li foil. The CF scaffold possessed a low resistance of 0.72 Ω cm^−2^, attributing to the intertwined bundle fibers and continuous fiber conductive networks. During the plating of Li, the porous fibers can accommodate Li in their interspace and thus lead to a relieved volume change of Li (Fig. 2F). More importantly, the lithophobic CFs with nonlithiophilic oxygenated functional groups on the surface promoted the deposition of Li far away from the anode/separator interface and thus achieved a favorable Li-free anode surface even after 30 mAh cm^−2^ of Li plating (Fig. 2F). COMSOL Multiphysics simulation in Fig. S1 provides substantiation for the above conclusion. The concentration distribution of Li ion was re-regulated due to the conductive CF scaffold at the top surface that can be coupled with inner electric field for achieving a uniform distribution of Li-ion flux. Meanwhile, the current density vector mainly pointed to the underlying Li foil, attributing to the lithophobic feature of the CF scaffold. This facilitated the favorable deposition of Li on the underlying Li foil with lower nucleation barrier and thus formed Li-free anode/separator interface for safer Li metal anodes.

To provide a visual monitoring of the Li plating behaviors, we conducted an in situ optical microscopy test to monitor the electrochemical plating behavior of Li on Li/CF anodes in real time. It can be seen in Fig. 2G and Movie S1 that the bare Li foil exhibited an increasing thickness toward both sides of the cross-section. Meanwhile, some dendritic Li appeared and gradually accumulated as extending the plating time. By contrast, Fig. 2H and Movie S2 show that the Li/CF anodes exhibited fundamentally different Li plating behavior. The lithophobic CF scaffold guided a directional Li-ion transport across the porous CF scaffold and finally deposited onto the underlying Li foil, which led to the desired “one-way” growth of Li metal toward CF scaffold (i.e., the right marginal line moved to the CF direction). As such, Li dendrite issue could be greatly relieved due to the favorable deposition path of Li, and meanwhile, the CFs could also be used as a porous skeleton to accommodate Li inside their interspace, which readily relieves the volume change of anode during charge–discharge cycles.

**Fig. 3.**
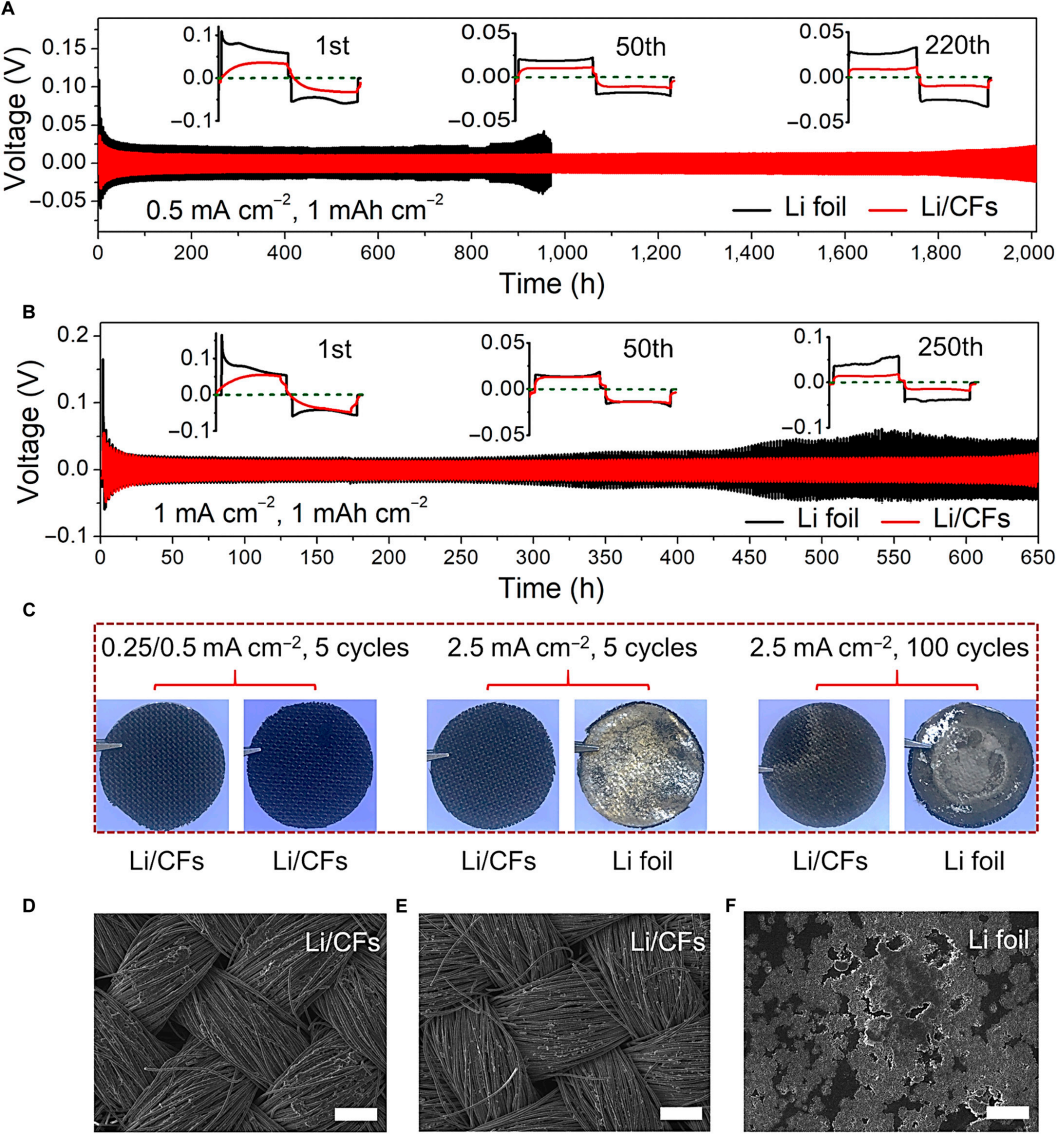
Cyclic stability of symmetric cells. Symmetric cell performances of Li-foil and Li/CF anodes at the current densities of (A) 0.5 and (B) 1 mA cm^−2^. (C) Photographs of Li-foil and Li/CF anodes after 5/100 cycles at 0.25/0.5/2.5 mA cm^−2^, respectively. SEM images of the Li/CF anodes after 5 cycles at (D) 0.5 and (E) 2.5 mA cm^−2^. (F) SEM image of the Li-foil anode after 5 cycles at 2.5 mA cm^−2^. Scale bars: 200 μm.

By assembling symmetric cells with a pair of Li/CF anodes, we measured the long-term cyclic stability with using bare Li foil as a comparison. As shown in Fig. 3A, the voltage curves of the Li/CF anodes demonstrated a 2-stage electrodeposition process of Li, i.e., Li ions were first intercalated into CFs to form LiC*_x_* complex (such as LiC_6_), followed by a Li metal deposition process. At the first cycle, the Li/CF anodes exhibited a lower overpotential of ~33 mV than that of the bare Li-foil anodes (~59 mV) at 0.5 mA cm^−2^, indicating the reduced Li nucleation/deposition barriers with using the CF scaffold. As the cycle continued, the Li/CF anodes demonstrated a lower-voltage hysteresis of ~9.6 mV and maintained stable even after 2,000 h (500th cycle). On the contrary, the bare Li-foil anodes exhibited a marked voltage fluctuation starting from 950 h (220th cycle), indicating the dendrite-induced battery failure. As the current density increased to 1 mA cm^−2^, the Li/CF anodes still maintained a high cyclic stability (~650 h) with a small polarization voltage of ~17 mV, whereas the bare Li-foil anodes showed a sharp oscillation occurred after only 300 h (Fig. 3B). To examine the ability of the Li/CF anodes to operate under more stringent conditions, we further measured the cyclic stability of symmetric cells at a higher current density of 5 mA cm^−2^ and larger capacities of 3.5 and 5 mAh cm^−2^, respectively. As shown in Fig. S2, the Li/CF anodes could still afford longer cyclic stability with increasing the current density and capacity as compared to the Li-foil anodes, indicating the high-rate and long-endurance capability of the Li/CF anodes to be used in future LMBs.

To investigate the mechanism of the improved cyclic stability by using the Li/CF anodes, we measured the surface morphologies of different anodes after cycling at fixed current densities (0.25/0.5/2.5 mA cm^−2^). As shown in Fig. 3C to E, the Li/CF anodes showed Li-free top surfaces after 5 cyclic tests at different current densities. Similar results can be also observed with the extended Li plating/stripping to 100 and 200 cycles (Fig. 3C and Fig. S3). The Li-free top surface suggests a remarkable improvement in the safety of the Li metal anodes, attributing to the reduced probability of dendric Li growth at the anode/separator interface. Moreover, the highly conductive CFs can be also coupled with the inner electric field to achieve a highly homogeneous Li-ion flux and thus resulted in an even deposition of Li. By contrast, the Li-foil anodes showed a cluttered top surface with an amount of mossy Li (Fig. 3C and F, and Fig. S3), indicating an irregular Li-deposition behavior on bare Li foil caused by a high local current density with using the planar structure.

**Fig. 4.**
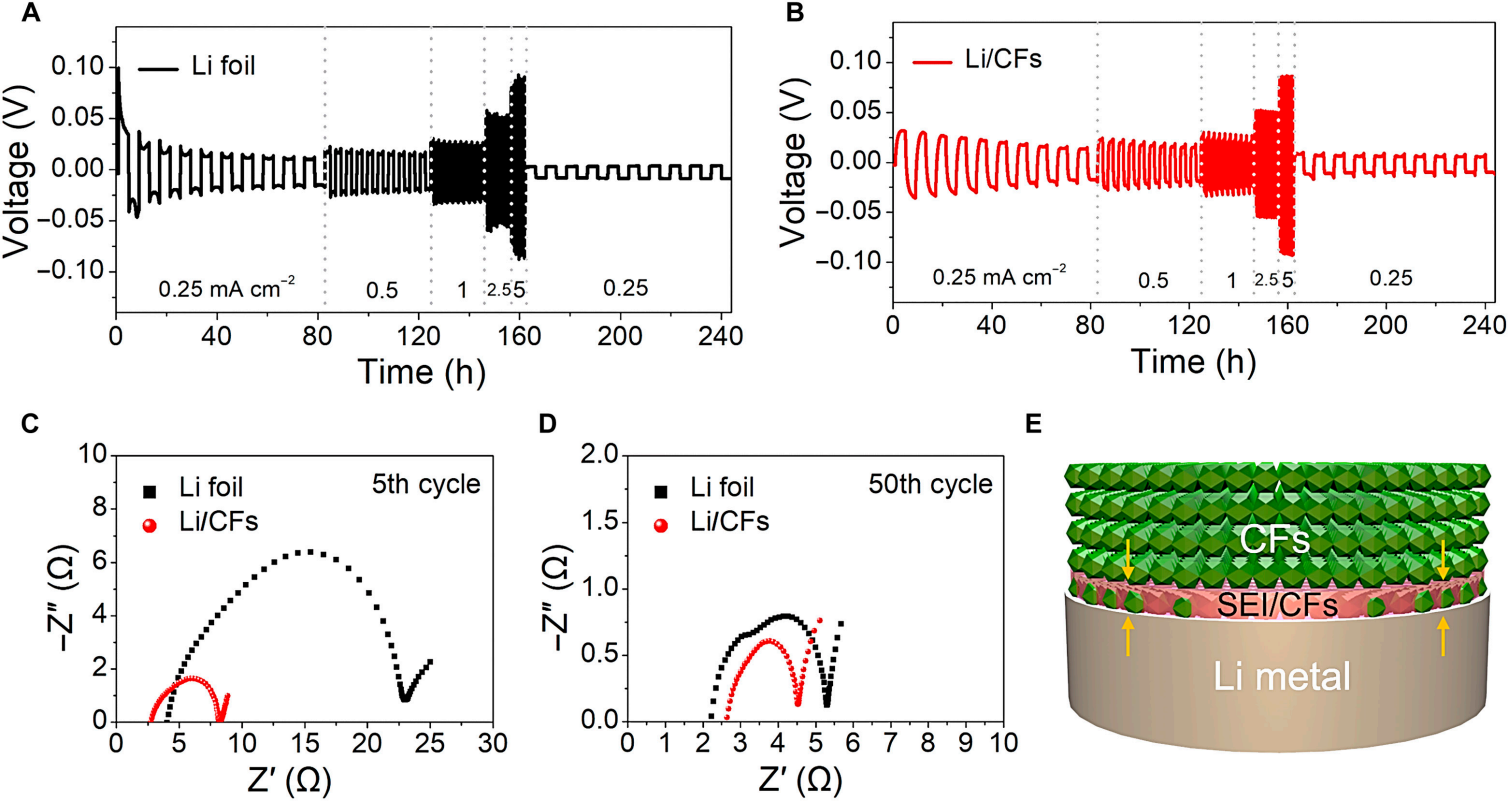
Electrochemical properties of the Li/CF anodes. Rate properties of (A) Li-foil and (B) Li/CF anodes. Nyquist plots of electrodes after cycling for (C) 5 and (D) 50 cycles, respectively. (E) Schematic illustration of the reinforced SEI using the Li/CF anode supported by CF skeleton.

To further prove the electrochemical property of the Li/CF anodes, we tested the rate capability of electrodes by assembling symmetric cells. As shown in Fig. 4A and B, a similar voltage hysteresis was presented for the Li-foil and Li/CF anodes with increasing the current densities from 0.25 to 2.5 mA cm^−2^. However, as the current density further increased to 5 mA cm^−2^, the Li-foil and Li/CF anodes demonstrated completely different voltage profiles. The marked voltage fluctuation of the Li-foil anodes indicates that an unstable SEI formed on the anode surface [37]. This stimulated the formation of excessive SEI and thus led to more consumption of electrolyte and Li metal for SEI reconstruction. By contrast, the Li/CF anodes exhibited an extremely stable voltage profile, indicating an improved stability of the SEI layer. As a proof of concept, we tested the resistance change of the electrodes at the 5th and 50th cycles using electrochemical impedance spectroscopy (Fig. 4C and D). Meanwhile, an equivalent circuit was established in Fig. S4 with the corresponding parameter data listed in Table S1. As the cycling increased from 5 to 50 cycles, the Li/CF anodes showed gradually decreasing interfacial resistance (*R*_ct_) from 3.31 to 1.67 Ω, which are lower than that of the Li-foil anodes (3.71 Ω after the 5th cycle and 2.38 Ω after the 50th cycle). This implies an improved interfacial affinity to Li and a reinforced SEI layer with faster reaction kinetics of Li stripping/plating by using the CF scaffold [46,47]. By covering a layer of CF on Li foil, we believe that the as-designed Li/CF anode exhibited a unique SEI configuration that has been immersed into interpenetrating CF skeleton on the Li-foil surface (Fig. 4E). Therefore, unlike the commonly used Li metal anodes, the SEI layer in the Li/CF anode is extremely stable due to the strong support by surrounding carbon fibers and thus can endure more severe electrochemical environments (e.g., higher current density and larger areal capacity) during battery cycling.

**Fig. 5.**
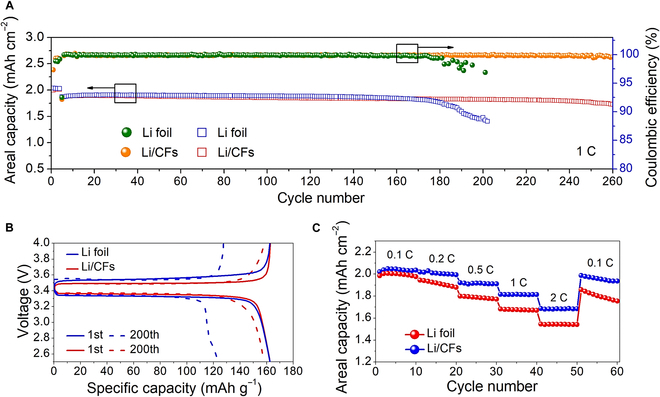
Cyclic tests of LMBs with the Li/CF anodes. (A) Cyclic performance of LMBs with the Li-foil and Li/CFs anodes. (B) Voltage-capacity curves of LMBs at the 1st and 200th cycles. (C) Rate properties of LMBs under various current densities.

In order to explore the further applications of the superior Li/CF anodes, LMBs were assembled by using LiFePO_4_ (LFP) as the cathode to assess the long-term cyclic stability of full cells. The areal loading of the LFP cathode was 11.52 mg cm^−2^, which supported a high areal capacity of 1.872 mAh cm^−2^. As shown in Fig. 5A, the battery with the Li/CF anode delivered a high capacity retention of ~92.2% after 260 cycles with a high average CE of ~99.87%. However, the full cell with the bare Li-foil anode showed an obvious capacity decay at 180 cycles. The profitable results of the Li/CF cell are mainly ascribed to the conductive and lithophobic CF scaffold, which contributed an extremely stable SEI and dendrite-free Li anode. Figure 5B shows the voltage-capacity curves of LMBs with the Li-foil and Li/CF anodes. The battery with the Li/CF anode showed a lower voltage polarization as compared to the Li-foil anode at the 1st cycle, and became more prominent with the increased charge–discharge tests to the 200th cycle. Impressively, the battery using the Li/CF anode held an outstanding capacity retention of ~99.99% per cycle, i.e., retain ~97.3% capacity after 200 cycles. Rate properties of LMBs were also investigated at variety current densities (Fig. 5C). The battery with the Li/CF anode exhibited much higher rate capacities (2.04, 2.01, 1.91, 1.81, and 1.68 mAh cm^−2^) than that of the Li-foil anode (2.00, 1.91, 1.79, 1.68, and 1.54 mAh cm^−2^) at 0.1, 0.2, 0.5, 1, and 2 C, respectively. The excellent cyclic stability and rate capability further manifested the superior Li/CF anode configuration for long-life and safe LMBs.

**Fig. 6.**
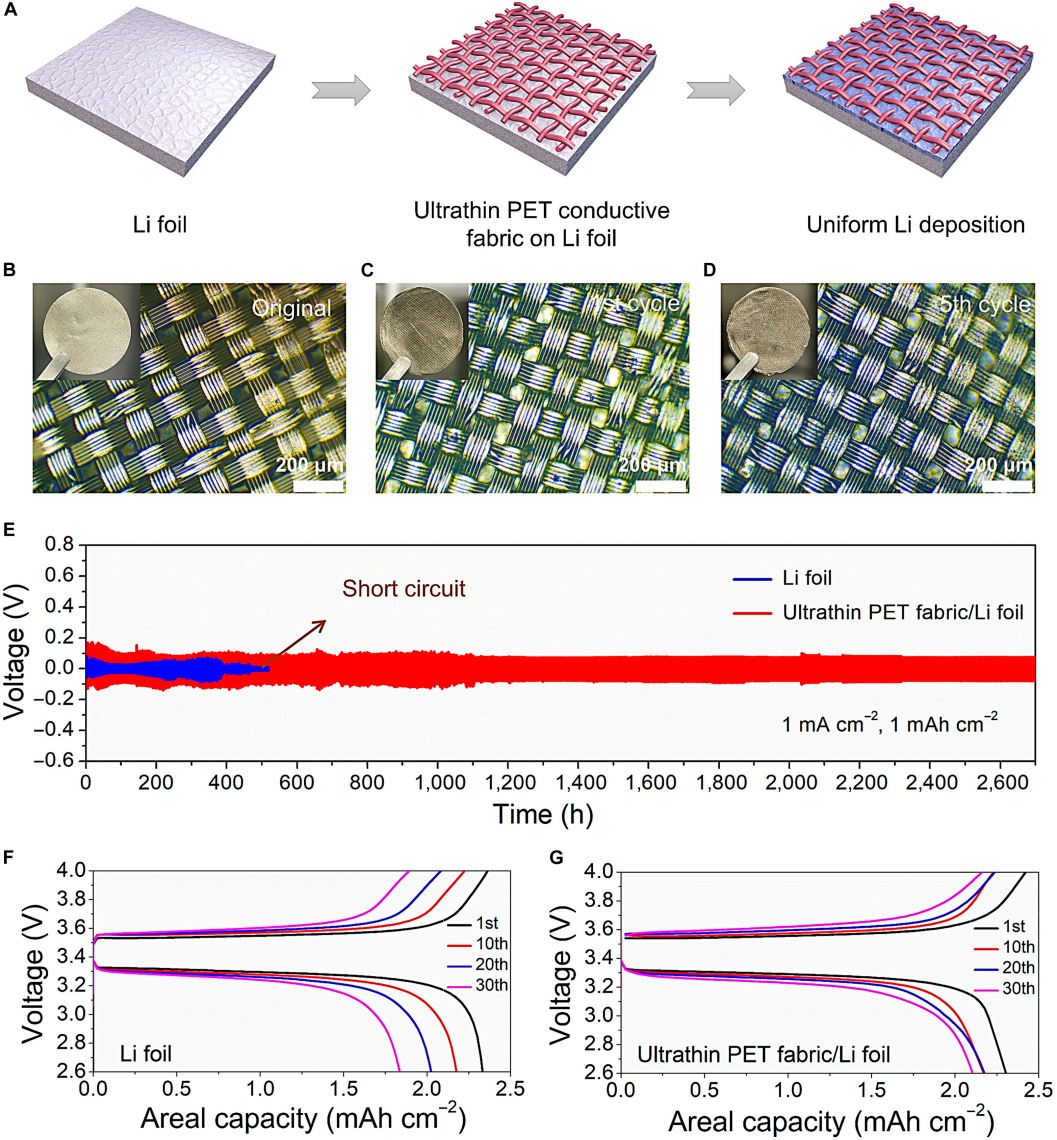
The universal anode structure extends to an ultrathin/ultralight PET conductive fabric (thickness: 20 μm, weight: 2.79 mg cm^−2^). (A) Uniform Li deposition regulated by the ultrathin/ultralight PET conductive fabric. Optical microscope images of the PET conductive fabric/Li-foil anodes after (B) 0, (C) 1, and (D) 5 Li plating/stripping cycles. (E) Symmetric cell properties with Li-foil and ultrathin PET conductive fabric/Li-foil anodes. Charge–discharge curves of LMBs with (F) Li-foil and (G) ultrathin PET conductive fabric/Li-foil anodes at the 1st, 10th, 20th, and 30th cycles.

In order to verify the feasibility of the conductive/lithophobic scaffold for high-energy Li battery application, we further demonstrated an ultrathin (thickness: 20 μm) and ultralight (weight: 2.79 mg cm^−2^) polyethylene terephthalate (PET) conductive fabric with Cu/Ni coating to guide a homogeneous Li deposition process (Fig. 6A). As shown in Fig. 6B to D, no Li metal appeared on the surface of the PET conductive fabric before and after the first and fifth plating/stripping cycles of Li, indicating the super-safe anode/separator interface with using the ultrathin PET conductive fabric on Li foil. Figure 6E shows the cyclic stability of symmetric cells with the PET conductive fabric/Li-foil anodes. The longer cyclic lifetime of over 2,600 h indicated the outstanding superiority of the PET conductive fabric/Li-foil anodes as compared to the bare Li foil that occurred short circuits before 400 h, which was mainly attributed to the favorable Li-free anode/separator interface. Figure 6F and G shows the charge–discharge curves of LMBs with the Li-foil and ultrathin PET conductive fabric/Li-foil anodes at different cycles. Compared with the Li-foil anode, the battery with the ultrathin PET conductive fabric/Li-foil anode demonstrated a lower capacity-decay rate, indicating the advantage of utilizing the ultrathin PET conductive fabric on Li foil. As a result, our conductive/lithophobic scaffolds are universal for different conductive films and thus applicable for high-energy LMB systems.

## Discussion

In summary, a reverse strategy of constructing stable Li metal anodes by using a highly conductive and lithophobic CF scaffold was proposed to achieve a Li-free anode/separator interface. Essentially, the lithophobic CFs induced a favorable Li-free anode surface and thus a remarkable improvement of the safety of LMBs. Meanwhile, the high conductivity of the CF scaffold provided a strong coupling with inner electric field for achieving a highly homogeneous Li-ion flux and thus a uniform deposition process of Li. The resulting Li/CF anodes exhibited ultralong cycle life (>2,000 h at 0.5 mA cm^−2^) with a relieved volume change and reinforced SEI. The full cells with the Li/CF anodes delivered an outstanding capacity retention of ~97.3% after 200 cycles. In addition, the Li-free anode/separator interface can be also achieved by using an ultrathin and ultralight PET conductive fabric, suggesting the advantages for future high-energy and super-safe LMBs.

## Materials and Methods

### Fabrication of the Li/CF anodes

Commercially available CFs (WOS1009, CeTech Co., Ltd) were treated with concentrated H_2_SO_4_/HNO_3_ (3:1 v/v) during sonicating at 80 °C for 4 h and then immersed in deionized water to remove the residual acid before dried at 80 °C. This procedure removed most of the impurities in CFs and thus could greatly reduce side reactions during battery cycling. For the Li/CF anodes, the acidified CFs with the size of 2 cm^2^ was placed on the surface of Li foil with the same area to serve as the conductive and lithophobic CF scaffold.

### Battery assembly

For assembling symmetric batteries, 2 same Li/CF anodes were used as the electrodes, while 1 M LiTFSI in 1,2-dimethoxyethane/1,3-dioxolane (1:1 v/v) with addition 2 wt% LiNO_3_ was used as the electrolyte. For assembling LMBs, Li/CF was used as the anode (area size: 2 cm^2^), LFP as the cathode (area size: 1 cm^2^), Celgard 2500 as the separator (area size: 3 cm^2^) and 1M LiPF_6_ in ethylene carbonate/dimethyl carbonate (1:1 v/v) as the electrolyte. In order to compare the performance between Li-foil and Li/CF anodes, an oversized electrolyte dosage of 40 μl cm^−2^ was used. For fabricating PET conductive fabric/Li-foil anode, commercially available PET conductive fabric with Cu and Ni coating was placed on the surface of Li foil to serve as the conductive and lithophobic scaffold. As the comparison, bare Li-foil anodes were used to assemble symmetric/full batteries by using the identical conditions as the Li/CF anodes. All batteries were prepared in an Ar-filled glovebox at ambient temperature.

### Material and electrochemical characterizations

The surface morphologies of samples were conducted with a VEGA3-TESCAN field-emission scanning electron microscope. For in situ optical imaging, a battery sample slot was employed with 2 terminal electrodes connected to battery testing systems. Electrochemical impedance spectroscopy was measured using a CHI660e electrochemical workstation. The CE of LMBs was calculated with the formula of CE_each cycle_ = Discharge capacity/Charge capacity. The average CE can then be obtained after equalizing the CE_each cycle_ to every cycle. The cyclic stability, CE, and rate properties of batteries were obtained at room temperature by using the NEWARE battery testing systems.

## Data Availability

All data needed to evaluate the conclusions in the paper are present in the paper and/or the Supplementary Materials.
