# Supplementary material for: Constructing Lithium-Free Anode/Separator Interface via 3D Carbon Fabric Scaffold for Ultrasafe Lithium Metal Batteries
Source: Research (Wash D C). 2023 Nov 8;6:0267. doi: 10.34133/research.0267 (PMC10907015; doi:10.34133/research.0267)
Supplement: Supplementary 1 — Figs. S1 to S4 Table S1 [file research.0267.f1.pdf]

Supporting Information

Constructing Lithium-Free Anode/Separator Interface via 3D Carbon Fabric Scaffold for Ultra-Safe Lithium Metal Batteries

Dongdong Li, Shengchen Yang, Zijian Zheng, and Wen-Yong Lai**

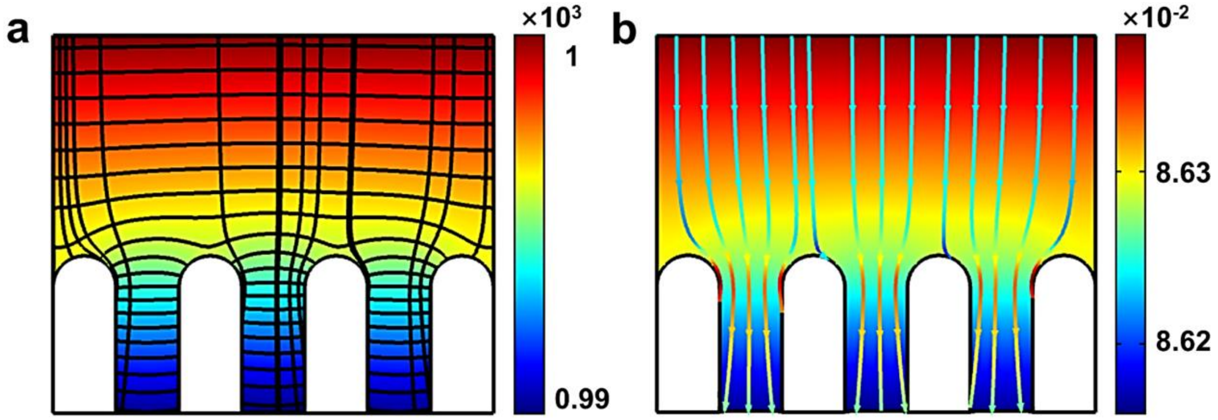

Figure S1. COMSOL Multiphysics modeling of (a) the concentration distribution of Li ion and (b) the current density on the Li/CF anode. In the modeling, the top boundary was defined as the cathode, while two bottom boundaries (i.e., the upper CFs and lower Li foil) were defined as the anodes. The initial Li-ion concentration was set as 1 mol L^{-1} with the diffusion coefficient of $2.93e^{-10}$ m^2 s^{-1}.

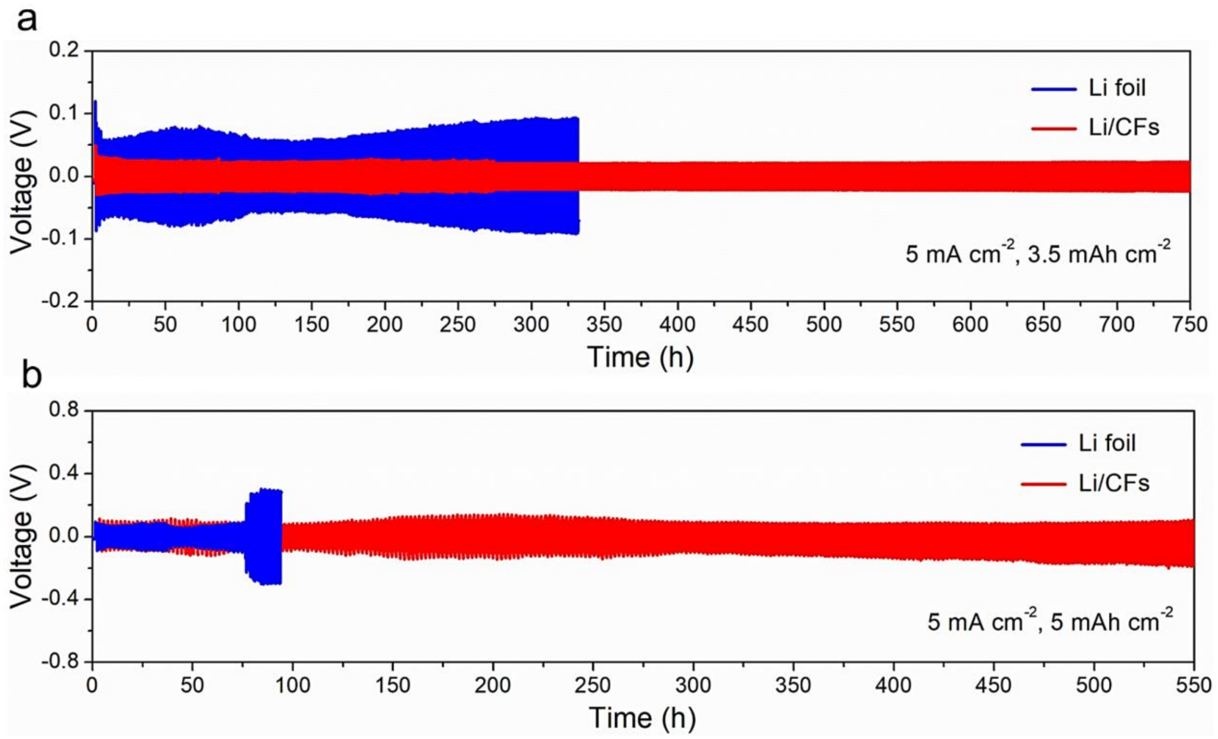

Figure S2. Symmetric cell performances of the Li-foil and Li/CF anodes at a) the current density of 5 mA cm^{-2} and the cyclic capacity of 3.5 mAh cm^{-2}, and b) the current density of 5 mA cm^{-2} and the cyclic capacity of 5 mAh cm^{-2}.

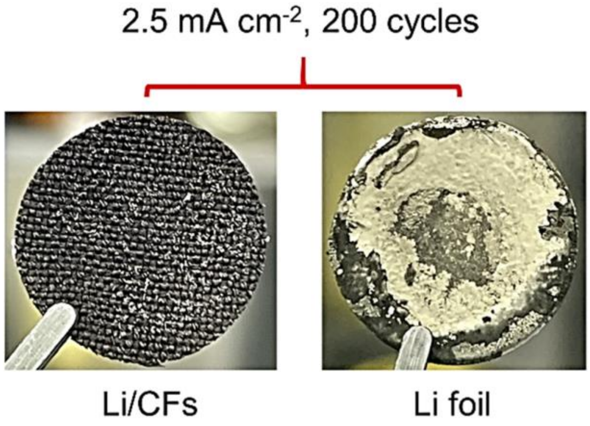

Figure S3. Photographs of the Li-foil and Li/CF anodes after 200 cycles at 2.5 mA cm^{-2}.

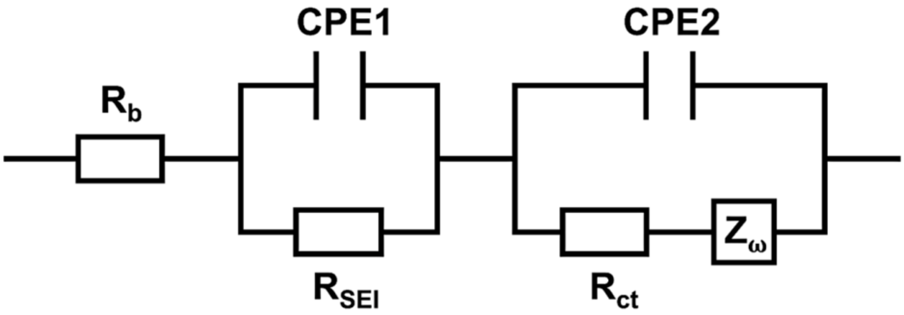

Figure S4. Equivalent circuit for Nyquist plots in Figure 4(c) and 4(d).

Table S1. Electrochemical impedance fitted parameters for Figure 4(c) and 4(d)

		Li foil			Li/CFs	
Cycle number	R_b	R_{SEI}	R_{ct}	R_b	R_{SEI}	R_{ct}
5	5.64	20.58	3.71	2.94	1.87	3.31
50	2.63	6.26	2.38	2.81	2.33	1.67
